# Supplementary material for: A detailed sensitivity analysis identifies the key factors influencing the enzymatic saccharification of lignocellulosic biomass
Source: Comput Struct Biotechnol J. 2024 Jan 26;23:1005–15. doi: 10.1016/j.csbj.2024.01.006 (PMC10900831; doi:10.1016/j.csbj.2024.01.006)
Supplement: MMC — Absolute value of the difference between the saccharification time-courses simulated for a specific set of input parameters and for the test sample versus the enzyme reaction rate of EG: A, B, and C; CBH: D, E, and F; and BGL: G, H, and I. The colour-code indicates: in A, D, and G: the relative position of the simulated saccharification curve with respect to the one of the test sample, as defined in the inset of Fig. 3B; in B, E, and F: the inhibition binding affinity of glucose to the respective cellulase; and in C, F, and I: the initial number of cellulase enzymes of the respective type. [file mmc1.pdf]

SUPPLEMENTARY MATERIAL

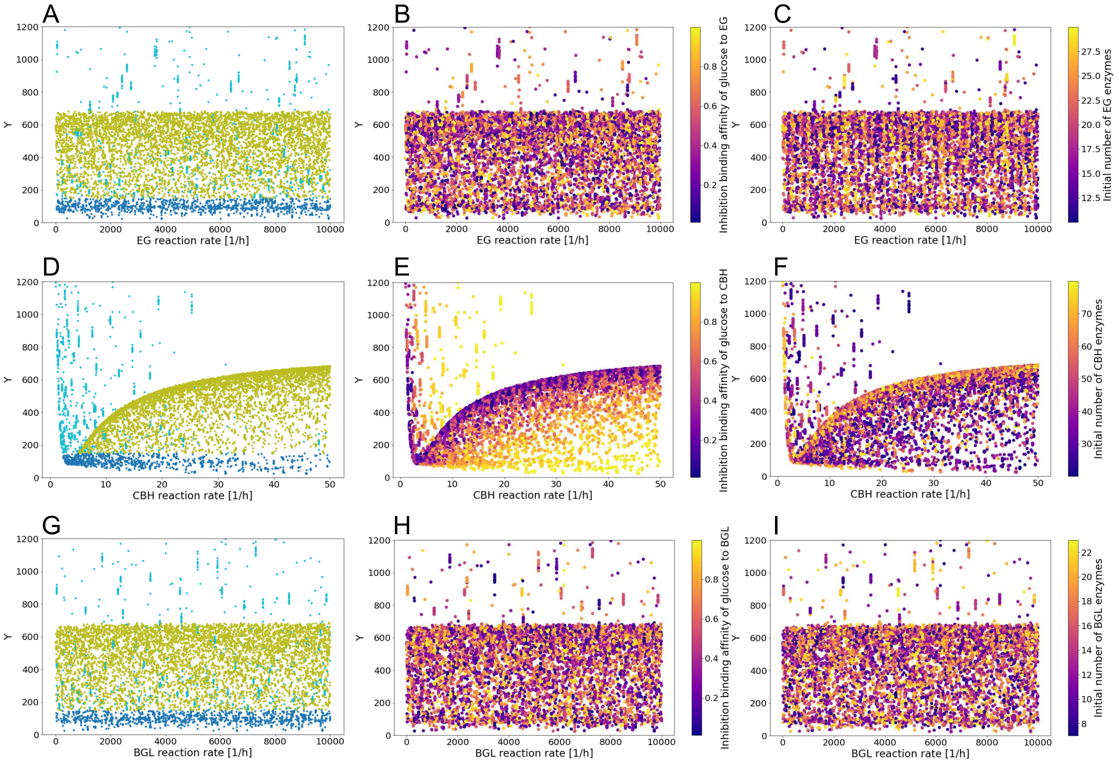

Figure 8: Absolute value of the difference between the saccharification time-courses simulated for a specific set of input parameters and for the *test sample versus* the enzyme reaction rate of EG: A, B, and C; CBH: D, E, and F; and BGL: G, H, and I. The colour-code indicates: in A, D, and G: the relative position of the simulated saccharification curve with respect to the one of the *test sample*, as defined in the inset of figure. 3B; in B, E, and F: the inhibition binding affinity of glucose to the respective cellulase; and in C, F, and I: the initial number of cellulase enzymes of the respective type.
